# Supplementary figures and images for: Effects of woody plant encroachment on abundance of multiple tick species in the U.S. Great Plains
Source: PLoS One. 2025 Oct 15;20(10):e0332832. doi: 10.1371/journal.pone.0332832 (PMC12527199; doi:10.1371/journal.pone.0332832)

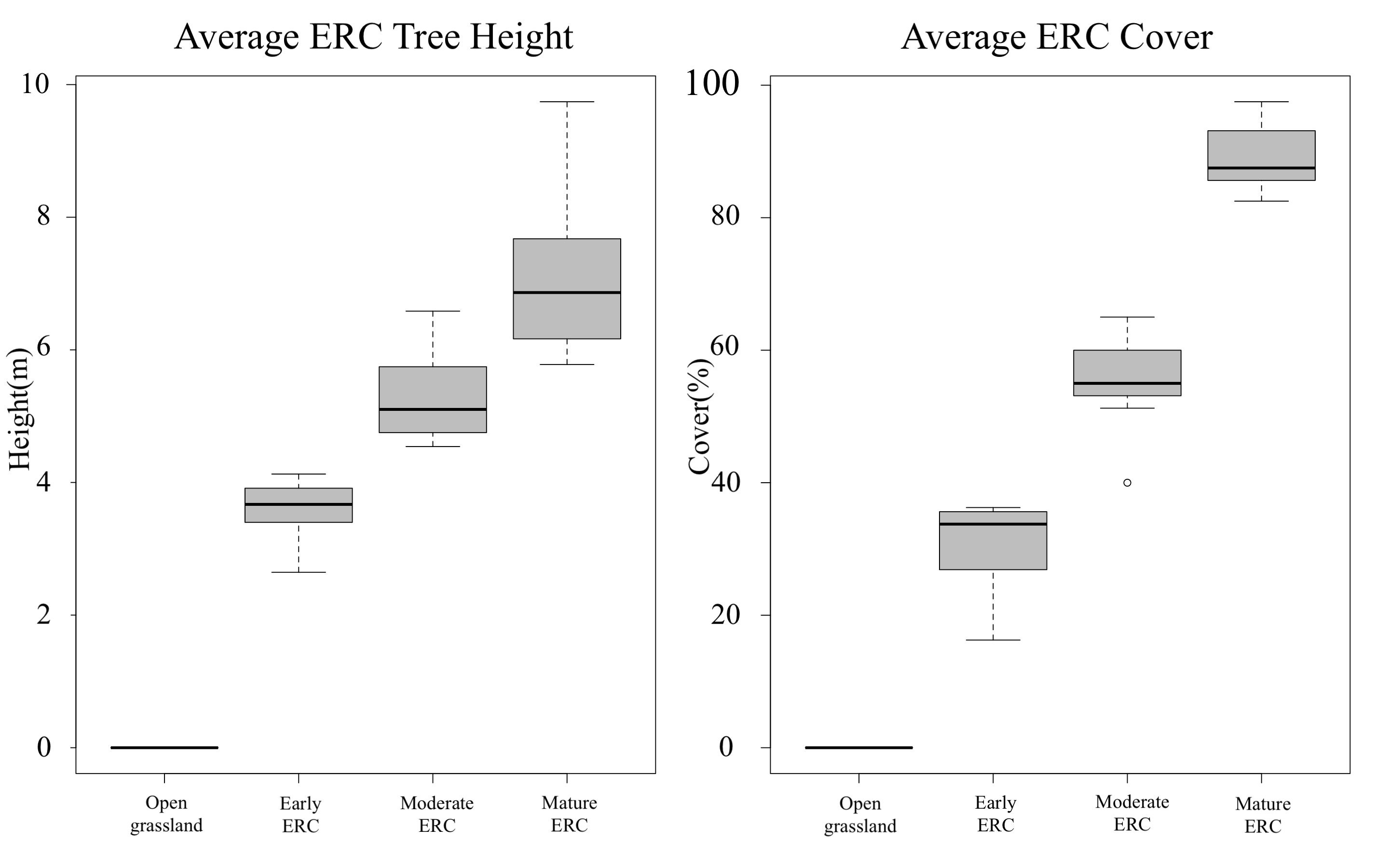

Supplement: S1 Fig — (TIFF) [file pone.0332832.s001.TIFF]
