# Supplementary material for: Effects of woody plant encroachment on abundance of multiple tick species in the U.S. Great Plains
Source: PLoS One. 2025 Oct 15;20(10):e0332832. doi: 10.1371/journal.pone.0332832 (PMC12527199; doi:10.1371/journal.pone.0332832)
Supplement: Attachment — Submitted filename: Propst_PLOSONE Respone to Reviewers.docx [file pone.0332832.s008.docx]

**Response to Reviewers:**

**We are grateful for the time that the reviewers have put into making our manuscript better. We have addressed each concern (see bold-face responses below each comment) and hope you find our improved manuscript acceptable for publication**

**Reviewer #1**:

Methods
Statistical Analysis.
Is there a reason for using abundance for the tick drags rather than density of ticks?

**AUTHOR RESPONSE: We appreciate this question from the reviewer. We focused on the abundance of ticks collected because our main study questions pertained to a landscape-level spatial scale (i.e., how does a certain area with a specific concentration of ERC impact abundance of ticks that are actively (flagging) and passively (CO2) questing for hosts?). Because all flagging was carried out the same way using different transects for each visit across each study area over the two years, we have a good idea of the abundance of actively-seeking ticks in a given ERC site, which is then comparable with other sites. As we aren’t asking questions related to disease risk (ie. to humans), we didn’t feel that quantifying and analyzing density (ticks/meter) would have addressed our research questions as well as using abundance does.**

How are you accounting for the different times of the year that ticks are collected in your analysis?

**AUTHOR RESPONSE: Thank you for your question. The primary objective of this study was to assess how tick abundance varies across sites representing four distinct stages of ERC encroachment and across the entire period of seasonal activity for each tick species. To capture these broad patterns, we did not focus on seasonality but instead evaluated abundance across all seasons. Nevertheless, we realize that assessing seasonal variation is an important consideration, and we have added new text in the limitations section of the Discussion (Lines 510-517 in the revised document) describing how we did not consider seasonal variation, and how evaluating hypotheses related to seasonality of ERC effects would be a fruitful area for future research.**

Regarding AIC, do you low sample sizes, it might be more appropriate to report AICc, which corrects for bias associated with small sample sizes and is more accurate.

**AUTHOR RESPONSE: In response to this reviewer’s later comment related to the relatively small number of models compared, we have decided not to use AIC for the main analysis of the effect of ERC on tick abundance and instead we use ANOVA analyses and p-values linked with pairwise comparisons of group means using the emmeans package. Therefore, this comment is no longer relevant.**

Line 247, how are you defining greater support. Is this delta AIC > 2?

**AUTHOR RESPONSE: In response to this reviewer’s later comment related to the relatively small number of models compared, we have decided to not use AIC for the main analysis of the effect of ERC on tick abundance. Therefore, this comment is no longer relevant. Instead, we used ANOVA analyses and p-values to identify whether the effect of ERC stage on tick abundance was statistically significant.**

Line 249-254: At first read, I wasn’t sure what you mean by stage. I have to reread. Could you use the actual category (grassland, early ERC, moderate ERC and mature ERC. This would make the job of the reader easier.

**AUTHOR RESPONSE: We appreciate the concern of the reviewer to make our categories understandable to the general reader. In response to this comment, we have decided to revise (see lines 153-162 in the Methods) to label the 4 categories as: Grassland (control), Early ERC encroachment, moderate ERC encroachment, and mature ERC encroachment (and for shorthand in some tables and figures, we use Grassland, Early ERC, Moderate ERC, and Mature ERC). We also revised to note that these categories correspond to stages 1-4 as defined in another paper that is currently in press and that we believe will be published, and therefore cite-able, before this paper is published.**

Also you are considering the grassland as the the reference stage or the control.

**AUTHOR RESPONSE: Grassland sites contained no ERC trees at the time of sampling; however, as we cannot guarantee that ERC encroachment had not already begun in grassland sites (in the form of ERC seeds deposited in the seedbank or on the ground), we think it is safer to refer to these as control sites and not reference sites (since reference sites generally imply pristine or idealized conditions, which in our case would mean grasslands free of any influence of ERC or its seeds). We have revised in the study design section to clarify that we considered open grassland sites to be our control treatment.**

I’m not sure what are you running separate models with each of the other stages of ERC encroachment as the reference for the intercept. To perform post comparisons, it would be more appropriate to use emmeans package as this would adjust the p-values for multiple comparisions?

**AUTHOR RESPONSE: Thank you for bringing this to our attention. As noted in other comments, we have revised to use an ANOVA and p-value testing approach for all of our analyses, now also linked with use of the emmeans package to conduct a single set of pairwise comparisons for each response variable. This allowed us to run a single model for each response variable and to account for the potential biasing effect of multiple statistical comparisons.**

Results
Table 2: I’m sure what you are using AIC when there are only two models to compare, one of which is the null. Usually you would have multiple predictors that you compare. In doing this, it would make more sense to just report the p-values.

**AUTHOR RESPONSE: We have addressed this comment by changing to use ANOVA analyses and p-values to assess the significance of the effect of ERC stage for each tick abundance response variable. Also as noted above, we now make comparisons between all group means for each analysis by using the emmeans package and post hoc pairwise comparisons. As a result of these changes, Table 2 is no longer needed**

Figures: for the X axis, instage of putting Stage 1,2,3 can you use “Open grassland”, “early ERC”, etc.

**AUTHOR RESPONSE: Although we have used these stage identifiers in previously published papers for the same study system, we appreciate the reviewers concern, and we have decided to revise to label the 4 categories as: Grassland (control), Early ERC encroachment, moderate ERC encroachment, and mature ERC encroachment (and for shorthand in some tables and figures, we use Grassland, Early ERC, Moderate ERC, and Mature ERC). We also revised to note that these categories correspond to stages 1-4 as defined in another paper that is currently in press and that we believe will be published before this paper is published.**

When describing the results, can you put it in context, instead of just stating the beta values, transform this into a percentage change. For instance in lines 318-319, based on the beta values, and if you are using a log link, the beta value can be convert indicates that "A. americanum was about 5 times as abundant in stage 2 as in stage 1," or ~400% more abundant, relative to stage 1."

**AUTHOR RESPONSE: We agree that statements about the magnitudes of differences between group means can add valuable context about the ecological significance of statistically significant differences. To address this comment, we have added such statements (e.g., at Lines 315-317 in the Results) about quantitative differences between tick abundances in different stages (particularly between grasslands and early ERC encroachment sites, which was the most frequent statistically significant difference that we documented).**

I’m not sure how you got the letter values for the figures.

**AUTHOR RESPONSE: As we had described in the captions for the bar graphs, letters shared among groups represent groups that are not significantly different from each other and letters that were different between groups indicate groups that are significantly different from each other. This is a standard convention across many scientific fields, as evidenced by the large number of online references and tutorials about this topic (here:** [**https://www.google.com/search?client=firefox-b-1-d&q=letters+on+bar+graphs+to+indicate+significant+differences**](https://www.google.com/search?client=firefox-b-1-d&q=letters+on+bar+graphs+to+indicate+significant+differences)**). If the journal’s editorial team has guidance or opinions on how better to present these graphs, we would of course defer to their recommendations.**

What is a coefficient analysis? See above comments about using emmeans.

**AUTHOR RESPONSE: Thank you for catching this lack of clarity. We meant that we had used coefficients from model parameters for the ERC stage fixed effect to estimate stage group means. We have revised this section (now at Lines 264-271 in methods) to clarify and to update this description to match the new emmeans analysis which generates estimates of group means and statistical significance of differences between them.**

Throughout the manuscript, you have a tendency to have the same information (AIC values, beta values) in the text that is in a table. Choose one,

**AUTHOR RESPONSE: Due to the change in our analysis approach to use ANOVA linked with pairwise comparisons, the tables with repeated information have been deleted. All information is now presented in either text or figure format, but not both.**

Discussion.
The discussion is well written given the current results, I won’t provide speciific comments as it is unknown if it will change when the method/results change.

**AUTHOR RESPONSE: We appreciate the assessment of the reviewer. The changes made regarding the statistical analysis did not produce any major changes to the storyline. For all analyses, we have still found a significant difference in abundance between grassland (control) sites and the earliest stage of ERC encroachment, although some of the previously shown differences between the 3 stages with ERC trees are no longer significant (i.e., all 3 stages of ERC encroachment have greater abundance than grasslands, but the 3 stages of encroachment are not significantly different from each other). Thus, the major conclusion that even the earliest stage of ERC encroachment increases tick abundance remains unchanged. That said, we have made some minor revisions in the discussion to align with our updated analyses and results.**

Minor Comments
Line 168L misspelling “centeral”

**AUTHOR RESPONSE: Spelling corrected as suggested.**

Lines 179: be consistent with the date format, earlier, you put the month day and on this line you put day month.
Be consistent with numbers, when using numbers less than 10, spell out the entire word.

**AUTHOR RESPONSE: Date format has been changed to be more consistent. We have also revised to make sure we refer to numbers correctly throughout the paper, although we note that this is more of a copy editing issue and will defer to the journal editorial staff for guidance on such issues.**

Line 258-263: what there an actually analysis for this? Also, the tect could be a bit more concise “Measurements of ERC trees confirmed that our sampling captured distinct stages of encroachment: both tree height and horizontal percent cover increased progressively across stages. Average tree heights for stages 1–4 were 0 m, 3.6 m, 5.3 m, and 7.2 m, respectively, while average horizontal cover was 0%, 30.2%, 55.2%, and 89.3%.”

**AUTHOR RESPONSE: We appreciate this comment and your suggested rewording. To clarify, there was no formal analysis run on measurements of ERC tree height and horizontal percent cover. Our goal was not to make this analysis a centerpiece of the study, but rather, to make it clear that our sites in different stages had some real separation in terms of the tree characteristics that likely influence tick abundance. To better introduce that this was a descriptive analysis, we have revised the Methods section that describes these two metrics to note that this was a descriptive comparison (Lines 220-224). In the results section referenced by the reviewer, and in addition to incorporating the above text revision suggestions, we have also revised the lead-in text to remind the reader that these are descriptive comparisons of stage means for ERC tree height and horizontal cover.**

Line 276-267: can you combine the first two sentences. Maybe “ June 2024, with increased abundance in 2024 than 2023.”

**AUTHOR RESPONSE: Text was revised as suggested.**

Line 280: from instead of “form”

**AUTHOR RESPONSE: Spelling corrected as suggested.**

**Reviewer #2:**

L40: Mention results for Amblyomma maculatum

**AUTHOR RESPONSE: Thank you for this comment. We revised to add results for *Amblyomma maculatum* to the abstract.**

L56: Capitalize initial letters of words in names only when proper noun

**AUTHOR RESPONSE: Corrected as suggested.**

L68: “effects” should be “affects”

**AUTHOR RESPONSE: Corrected as suggested.**

L79: “Eastern redcedar” should be “eastern redcedar”; be consistent with the use of common names and capitalization of initial letters. My suggestion would be that the only words in common names to have an initial capitalized letter should be proper nouns e.g., Bourbon virus, West Nile virus, Bachman’s sparrow, otherwise, they should be, for example, white-tailed deer, white-footed mouse, etc. The alternative would be to capitalize each initial letter e.g., White-tailed Deer. But, it should never be White-tailed deer. Whichever you prefer, be consistent throughout the manuscript. Double check that all other common names are correctly written.

**AUTHOR RESPONSE: Thank you for catching this inconsistency with the use of common names and capitalization. We went through and corrected all common names to have consistent capitalization. Please also note that we will of course defer to the journal editorial staff on such formatting issues, should we reach the copy-editing stage for this paper,**

L88: Suggest inserting “those of” between “including” and “birds”

**AUTHOR RESPONSE: Text has been revised based on your comment.**

L120: The Methods are well presented, but could use additional detail in some areas. In particular, details about the trapping and flagging need to be included.

**AUTHOR RESPONSE: In response to the reviewer’s suggestion, we have added more details in regard to CO2 trapping.**

L133: If abbreviations are to be used, be consistent with their use. Here, be consistent with use of ERC v. eastern redcedar, and check throughout the manuscript. Also check WPE. Once defined, subsequent mentions of the phrase should be abbreviated.

**AUTHOR RESPONSE: We have gone through and changed all subsequent eastern redcedar to ERC and woody plant encroachment to WPE.**

L136-145: Consider presenting this information as a table

**AUTHOR RESPONSE: We have revised to add a table as suggested, including the first column showing listing the two broad land cover types we sought to identify (grassland and ERC tree cover) and the second column listing the specific cover categories included in the search for each broad land cover type.**

L179: Awkward wording, suggest rewording: “periods when tick activity averages lower than during early summer”

**AUTHOR RESPONSE: Reworded for clarity as suggested.**

L176-179: Be consistent with date formatting: April 1st – July 15th vs. 16 July – 31 Oct)

**AUTHOR RESPONSE: Text has been revised as suggested.**

L193: Is Ixodes abbreviated here as Ix. for a reason?

**AUTHOR RESPONSE: Thank you for catching this inconsistency. All abbreviations of *Ixodes* have been revised to be “I.”.**

L194: Elaborate on the CO2 traps and flagging methods with citations. Were traps commercially purchased or built? Give specifics about flagging. How long were traps left in place and was this consistent across site visits? Were transects linear?

**AUTHOR RESPONSE: We have added the requested details, including noting that transects for flagging were linear and that our flagging approach followed widely used CDC guidelines, as well as additional details about the construction and operation of CO2 traps.**

L200: Cite keys that were used.

**AUTHOR RESPONSE: Citations were added as suggested.**

L209-213: Can this be reworded for clarity? I think I understand what is being communicated, but it took a moment to interpret, especially L210

**AUTHOR RESPONSE: Thank you for this comment. Text has been reworded for clarity, specifically to describe that one tree was selected in each of five randomly chosen compass directions at each site…resulting in 5 trees/measurements per site and 15 total trees/measurements per site cluster (3 sites in each cluster with ERC trees times 5 trees per site = 15).**

L221: Could statistical comparisons not be performed on mean tree height and horizontal percent cover between encroachment stage categories?

**AUTHOR RESPONSE: Our goal was not to make this analysis a centerpiece of the study, but rather, to make it clear that our sites in different stages had some real separation in terms of the tree characteristics that likely influence tick abundance. While statistical analyses could have been run, we think that descriptive presentations and summaries of these measurements is enough to make the preliminary point that sites had different ERC characteristics. To better introduce that this was a descriptive analysis, we have revised the Methods section that describes these two metrics to note that this was a descriptive comparison (Lines 220-224). In the results section referenced by the reviewer, and in addition to incorporating the above text revision suggestions, we have also revised the lead-in text to remind the reader that these are descriptive comparisons of stage means for ERC tree height and horizontal cover.**

L228: Were larvae collected?

**AUTHOR RESPONSE: Larvae were not collected in this study. We revised the methods section to include this statement.**

L240: Spell out Akaike Information Criterion here, put abbreviation in parentheses

**AUTHOR RESPONSE: Text has been revised** **as suggested to spell out Akaike Information Criterion at first mention in the Methods section.**

L268-270: For consistency, abbreviate names of genera after the first mention of a species name

**AUTHOR RESPONSE: Text has been revised** **as suggested.**

L280: Change “form” to “from”

**AUTHOR RESPONSE: Spelling corrected** **as suggested.**

L284: Table 1 legend – for clarity, insert “(stage 1)” after “open grasslands”

**AUTHOR RESPONSE: In response to other reviewer comments, please note that we have changed the names for the four different stages. In the text, we now use grassland (control), Early ERC encroachment, moderate ERC encroachment, and mature ERC encroachment (and for shorthand in some tables and figures, we use Grassland, Early ERC, Moderate ERC, and Mature ERC). So, in this table, we have now revised to use the shorthand designations for each stage category.**

L308: Would it be meaningful to run this analysis for total Amblyomma americanum? (i.e., Amblyomma americanum adults + nymphs, with trapping method counts combined, as was done for Amblyomma maculatum and Dermacentor variabilis.) Consider including that if it would be meaningful.

**AUTHOR RESPONSE: This is an interesting observation and suggestion. While it is common practice to combine all life stages and collection methods in a single analysis, we believe the overall trend would remain unchanged (early, moderate, and mature ERC sites consistently show significantly higher *A. americanum* abundance compared to open grassland). Additionally, combining all life stages and collection methods would obscure the important nuances we observed in how different *A. americanum* life stages respond to ERC encroachment.**

L362: Insert “(stage 1)” after “grasslands”

**AUTHOR RESPONSE: See above response regarding the change of our stage category names, which we now reflect in revised table and figure labels as well.**

Discussion: The Discussion section is well written, and I appreciate the inclusion of the study’s limitations, especially the potential that trap placement could have biased results. The only weak part of the Discussion is the first paragraph. The language here could be adjusted to make references to trapping methods and stages more clear. It could also be helpful to split this into two paragraphs, with the first focusing summarizing the most important results, (briefly) reiterating the question and the work that was done, and the second explaining the details described in L372-378.

**AUTHOR RESPONSE: We appreciate the perspective of the reviewer regarding he first paragraph of the discussion. We see the first paragraph as place to summarize the main take-homes of the study after which each paragraph will unpack some of the information presented. As it all tells the same story, we feel it works better to keep it together. We have, however, done some editing to make the paragraph more focused on the main results.**

L368: Consider including in this first sentence a statement referencing that Amblyomma maculatum abundances were lower in the Stage 2-4 samples. This result is similarly important as those for the other two species.

**AUTHOR RESPONSE: Results for *A. maculatum* were added** **as suggested.**

L373: Suggest inserting “(overall)” after D. variabilis

**AUTHOR RESPONSE: Text has been revised** **as suggested.**

L375, 376: For clarity, suggest replacing “collected by trapping” to “collected in CO2-baited traps”

**AUTHOR RESPONSE: Changed “trapping” to CO2 traps” as this verbiage was used in methods section to describe the method.**

L398: Juniper, or redcedar?

**AUTHOR RESPONSE: Changed to “ERC”** **as suggested.**

L488: I would include in this initial sentence mention of your results for Amblyomma maculatum as well. It is certainly important that abundance was higher for Amblyomma americanum and Dermacentor variabilis in grasslands with woody plant enchroachment, but it is equally meaningful that other tick species, Amblyomma maculatum, was more abundant in native grasslands. Given that, I would also reword this first sentence a bit – rather than stating that this encroachment facilitates increased abundance of ticks, broadly, I think it would be more accurate to describe this is “some species of ticks”. It is a bit misleading without that caveat.

**AUTHOR RESPONSE: We agree that we should highlight this pattern for A. maculatum as well to avoid the perception of a bias towards us stating that ERC increases abundance of all tick species. On the other hand, it is well-known that *A. maculatum* is a grassland-inhabiting species, so the result for this species is unsurprising. We have revised in a couple locations in the Discussion to highlight this opposite pattern for *A maculatum*, but rather than adding extensive discussion about this expected result, we have used some shorthand text to note that this is a somewhat expected pattern given the known association of this species with more open vegetation cover types.**

L493: Would it be worthwhile to rerun your analysis with all three tick species combined? If that were done, you could generalize about ticks a bit more broadly.

**AUTHOR RESPONSE: This is an interesting observation and suggestion, but we note that there are two reasons why we did not do this analysis of total ticks. First, Lone Star ticks (*A. americanum*) comprised a very high percentage (98.8%) of our entire sample of ticks, so combining all species would result in analysis results that mirror the results for this species. Additionally, each species and life stage has different ecology (related to vegetation associations and host affinities), and by combining them, we would lose the nuance of each species and life stage (e.g., the above highlighted opposite association of *A. maculatum* with ERC encroachment). As a result of these factors, we have decided not to include an additional analysis based on total abundance across all tick species.**

L500: Should ERC be WPE?

**AUTHOR RESPONSE: We appreciate this question but in this case, it needs to be ERC.**

L509: Was anything known about the fire regime at the study sites? Consider including a note regarding this in the Methods or discussing as a limitation in the Discussion.

**AUTHOR RESPONSE: Thank you for highlighting the importance of fire regimes, although after considering this comment, we are not certain what limitation the reviewer is suggesting related to fire regimes. We acknowledge that we did not explicitly analyze or consider fire regimes as part of our analysis. However, we note that grassland sites have received regular disturbance by fire (that is why they are grasslands and not ERC-encroached areas) whereas ERC sites have not experienced recent fire (that is why they are no longer grasslands). We do not think this difference in fire regimes is a limitation, but is rather a crucial mechanism underlying the differences between these sites that leads to the differences in vegetation cover that form the focal point for our analysis. We think that adding some of this nuance about fire histories, and how they have led to the different types of sites that we studied, helps address this reviewer’s concern and should also be useful for readers who are not familiar with ours and similar study systems. We added a new paragraph in the study design section that discusses fire regimes as well as our assumptions (and in some cases known information, based on available fire histories) about our grassland and ERC sites. That description (at Lines 168-179) follows here:**

**“Regarding the fire regimes at our study sites, we note that throughout our study region, frequent disturbance by fire, including wildfire and prescribed fire (i.e., modern prescribed fire efforts for rangeland, wildlife, and ecosystem management, as well as historic use of fire by indigenous cultures for thousands of years) has historically maintained open grassland ecosystems (Twidwell et al. 2017, 2021). Suppression of fire on the landscape has allowed expansion of tree cover, including encroachment by ERC, a species that is readily top-killed by fire. Detailed fire histories for some of our site clusters (e.g., those on lands managed by Oklahoma State University for research purposes) confirm that prescribed fire has regularly occurred at grassland sites and has been excluded from sites containing ERC trees. For site clusters without fire histories available, we infer that fires have occurred at grassland sites because otherwise these sites would have begun transitioning to ERC cover; likewise, at sites with ERC encroachment, we infer that fires—or at least top-killing canopy fires—have been excluded for at least for as long as ERC trees have been established.”**

L511: “of some species” should be inserted after “increased tick populations”

**AUTHOR RESPONSE: Text has been revised** **as suggested.**
